# Controlled Release Fertilizers: A Review on Coating Materials and Mechanism of Release

**DOI:** 10.3390/plants10020238

**Published:** 2021-01-26

**Authors:** Dora Lawrencia, See Kiat Wong, Darren Yi Sern Low, Bey Hing Goh, Joo Kheng Goh, Uracha Rungsardthong Ruktanonchai, Apinan Soottitantawat, Learn Han Lee, Siah Ying Tang

**Affiliations:** 1 Chemical Engineering Discipline, School of Engineering, Monash University Malaysia, Bandar Sunway 47500, Selangor Darul Ehsan, Malaysia; dora.lawrencia@monash.edu (D.L.); see.wong@monash.edu (S.K.W.); darrenl333.dl@gmail.com (D.Y.S.L.); 2 Biofunctional Molecule Exploratory Research Group, School of Pharmacy, Monash University Malaysia, Bandar Sunway 47500, Selangor Darul Ehsan, Malaysia; goh.bey.hing@monash.edu; 3 College of Pharmaceutical Sciences, Zhejiang University, Hangzhou 31005, Zhejiang Province, China; 4 School of Science, Monash University Malaysia, Bandar Sunway 47500, Selangor Darul Ehsan, Malaysia; goh.joo.kheng@monash.edu; 5 National Nanotechnology Center, National Science and Technology Development Agency, Pathum Thani 12120, Thailand; uracha@nanotec.or.th; 6 Center of Excellence in Particle and Materials Processing Technology, Faculty of Engineering, Chulalongkorn University, Bangkok 10330, Thailand; apinan.s@chula.ac.th; 7 Novel Bacteria and Drug Discovery Research Group, Microbiome and Bioresource Research Strength, Jeffrey Cheah School of Medicine and Health Sciences, Monash University Malaysia, Bandar Sunway 47500, Selangor Darul Ehsan, Malaysia; 8 Tropical Medicine and Biology Platform, School of Science, Monash University Malaysia, Bandar Sunway 47500, Selangor Darul Ehsan, Malaysia; 9 Advanced Engineering Platform, School of Engineering, Monash University Malaysia, Bandar Sunway 47500, Selangor Darul Ehsan, Malaysia

**Keywords:** controlled release fertilizer, coating materials, release, mechanism, nutrients

## Abstract

Rising world population is expected to increase the demand for nitrogen fertilizers to improve crop yield and ensure food security. With existing challenges on low nutrient use efficiency (NUE) of urea and its environmental concerns, controlled release fertilizers (CRFs) have become a potential solution by formulating them to synchronize nutrient release according to the requirement of plants. However, the most significant challenge that persists is the “tailing” effect, which reduces the economic benefits in terms of maximum fertilizer utilization. High materials cost is also a significant obstacle restraining the widespread application of CRF in agriculture. The first part of this review covers issues related to the application of conventional fertilizer and CRFs in general. In the subsequent sections, different raw materials utilized to form CRFs, focusing on inorganic and organic materials and synthetic and natural polymers alongside their physical and chemical preparation methods, are compared. Important factors affecting rate of release, mechanism of release and mathematical modelling approaches to predict nutrient release are also discussed. This review aims to provide a better overview of the developments regarding CRFs in the past ten years, and trends are identified and analyzed to provide an insight for future works in the field of agriculture.

## 1. Introduction

The world population is forecasted to increase by one third or 2.3 billion in 2050, despite the slower growth rate compared to the last four decades [1,2]. As the population keeps blooming, food demand is also expected to rise. Fertilizers provide nutrients to plants and are often credited for the improvement in crop yield, which results in exponentially increased fertilizer use worldwide. Among the three macronutrients required (Nitrogen/Phosphorus/Potassium), nitrogen (N) is the most crucial and essential to plant growth, and urea is the most commonly used nitrogen-based fertilizer due to its high N content (46 wt.%) and its low cost [3,4,5]. However, it is prone to being lost due to poor absorbance by crops, contributing to economic decline and severe environmental concerns such as nitrate leaching, groundwater contamination, soil acidification, heavy metal contamination and eutrophication of freshwater bodies [6,7,8]. On the other hand, the periodic dosing of fertilizer also results in concentration fluctuations between ineffective and toxicity levels, which may affect the growth of certain agricultural species [9].

There has also been increasing interest in applying biocontrol agents such as *Streptomyces* bacteria [10] and phenazine-1-carboxylic acid (PCA) found in commercialized biopesticide formulations [11]. It was reported that *Streptomyces* could produce various bioactive compounds [12,13,14] and inhibit various phytopathogenic fungi [10], while active components such as PCA exhibited strong redox, conferring resistance oxidative stress in plant cells [11]. However, the successful application of these biocontrol agents remains a challenge due to wide variations in environmental conditions. Therefore, researchers are still actively looking for a better solution to improve the utilization efficiency of fertilizers and reduce adverse environmental impacts associated with their use.

Controlled release fertilizers (CRFs) have been extensively studied to provide a safer, more economical, and efficient way of administering nutrients as they are made available to the target at the desired rate or concentration level, hence sustaining the nutrients in the soil for a longer period [7,15,16]. This helps to improve nutrient use efficiency (NUE) by less frequent dosing and reduced environmental hazards by diminishing nutrient removal rate from the soil by rain or irrigation [17]. 

Commercial CRFs using polymer coatings are mostly made of thermoplastic resin such as polyolefin, polyvinylidene chloride and copolymers which cannot degrade easily in soil and can accumulate overtime. According to the International Fertilizer Association (IFA) [18], on the agreement of the new European Union (EU) Fertilizer Regulation, the EU is also working on biodegradability criteria for polymer coatings in CRFs. Therefore, the research focus has been shifted to the development of CRFs from environmentally friendly and safer materials that can provide better performance on controlling release rate. 

Therefore, this work aims to review the types of coating materials, preparation methods and release patterns of CRFs, discuss empirical and mechanistic approaches in modelling nutrient release and identify the future direction associated with the development and utilization of CRFs, through recent studies. 

## 2. Limitations of Conventional Fertilizers

**Figure 1:**
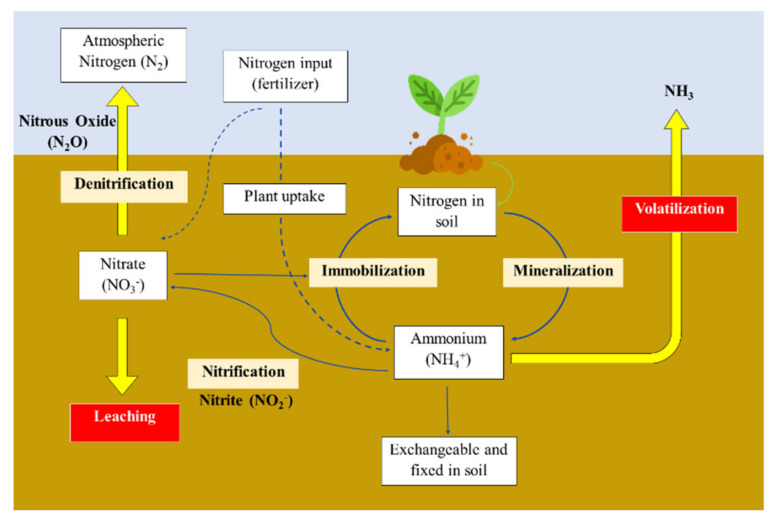
Nitrogen cycle in soil. Adapted from [23].

Direct administration of chemical fertilizers to plants was shown to have low utilization efficiency as only 30–35% of the nutrients are absorbed [3,4,19,20]. Urea, the most commonly used N-fertilizer, was reported to have NUE levels of only 50%, where 2–20% is lost through volatilization, 15–25% reacts with organic compounds in the soil and 2–10% is lost through leaching into water systems, leading to pressing environmental concerns [4,6]. Figure 1 shows a simplified nitrogen cycle in the soil and how naturally occurring nitrogen in the soil and fertilizer change from one form to another. Nitrogen in urea is converted by urease enzymes in the soil to ammonium through mineralization, then converted to nitrite and nitrate ions via the nitrification process. When the soil cannot retain the urea due to excessive water from irrigation or heavy rainfall, nitrate ions will leach to ground and surface water bodies. Consequently, high concentrations of nitrate ions in plants and drinking water could pose high risks to human health [6,8]. Besides water pollution, nitrogen is also lost through volatilization as N_2_ and N_2_O, through complete and incomplete denitrification processes, respectively [21]. Ammonium could also be lost as NH_3_ through volatilization. Nitrogen-based fertilizers have also been reported to be the source of N_2_O, which is the primary substance for worldwide ozone depletion in the 21st century [6,22].

## 3. Controlled Release Fertilizer (CRF)

**Figure 2:**
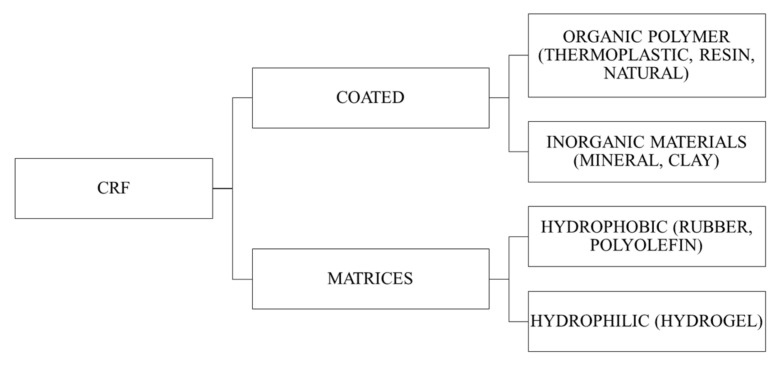
Simplified classification of controlled release fertilizers (CRFs).

Slow release fertilizer (SRF) and controlled release fertilizer (CRF) are often used interchangeably. SRF is known as “low solubility compounds with a complex/high molecular weight chemical structure that release nutrients through either microbial or chemically decomposable compound” [24], where CRF can be defined as “products containing sources of water-soluble nutrients, the release of which in the soil is controlled by a coating applied to the fertilizer” [25]. SRFs are generally classified into condensation products of urea-aldehydes, fertilizers with a physical barrier (coated or incorporated into matrix) and super granules. CRF is a subset of SRF which falls under the category of fertilizer with a physical barrier. The simplified classification of CRFs is presented in Figure 2.

Although the required nutrient release rate by CRF varies for each plant depending on the metabolic requirements of the crop for a specified period, the European Standardization Committee (CEN) Task Force has made some recommendations on the criteria of CRF such that the rate of nutrient release must be slower than conventional fertilizer, not more than 15% of the nutrients are released within 24 h, not more than 75% of the nutrients are released within 28 days and at least 75% of the nutrients are released within the stated release time [23]. 

Some other factors that are required from a CRF include cost-effectiveness, being environmentally friendly and sustainability. 

### 3.1. Advantages of CRF

The application of CRF can help to improve NUE and reduce nutrient loss, primarily through nitrate leaching and the volatilization of ammonia and nitrous oxides, which contribute to minimizing environmental pollution. It is also possible to decrease the fertilizer application rate by 20 to 30% of the recommended value to achieve the same yield [7,23]. This can provide economic advantages in terms of saving labor, time, and energy. In addition, when CRF releases nutrients at a desirable rate (preferably in a sigmoidal pattern), it contributes to agronomic safety by reducing the toxicity imposed to plants, especially seedlings [26,27]. This is because the conventional practice of chemical fertilizer application tends to result in the high local concentration of ions, which induces osmotic stress and causes damage to plants [26,27].

### 3.2. Disadvantages of CRF

There are still no standardized methods to determine the nutrient release rate from CRF in a reliable way. There is also a lack of correlation between data obtained from laboratory studies and the actual nutrient release rate in practical applications that can be made available to consumers [23]. In addition, CRFs have not always been compared to the best fertilizer management practices when reporting about their advantages [28]. Using a CRF such as Sulfur Coated Urea (SCU) in large quantities can increase soil acidity, while polymer-coated CRFs using synthetic materials may be difficult to degrade, which contributes to other forms of pollution. During the application of CRF, nutrients may continue to be released even in the absence of plants due to the tailing effect. The tailing effect occurs after 80–85% of the nutrients are released and the remaining nutrients are released in a prolonged manner [26]. Furthermore, the cost for manufacturing CRF today is still much higher compared to conventional chemical fertilizers, which restricts its widespread use in agriculture [23].

## 4. Coating Materials

Coating materials are typically made up of two categories, namely inorganic materials and organic polymers [23]. Inorganic materials include sulfur, bentonite, and phosphogypsum while organic polymers can be either synthetic polymers, such as polyurethane, polyethylene, alkyd resin, etc., or natural polymers such as starch, chitosan, cellulose, and others [23]. Additionally, recent studies show that organic materials such as biochar, rosin and polyphenol are being utilized [29,30,31]. Different combinations of these materials were explored to examine the effect on the release rate of urea and to determine their possibility as coating materials for CRFs. This section categorizes the materials into four groups, namely inorganic material-based, synthetic polymer-based, natural polymer-based and other organic materials.

### 4.1. Inorganic Material-Based Coatings

Most of the inorganic based coating materials are made from sulfur and minerals. Sulfur based coating CRF, SCU, was one of the earliest developed by Tennessee Valley Authority (TVA) in 1961. However, as the coating is typically damaged with imperfections, nutrient release cannot synchronize with plant requirements and results in immediate release when it comes into contact with water, termed as the “burst effect” [23]. Hybrid coatings of polymer and low cost sulfur (about 38.5–42% N, 11–15% S and less than 2% polymer sealant) were developed to overcome this problem, but despite some improvements, the “burst” and “lock-off” characteristics still persist [23]. 

Other low-cost sulfur based materials such as gypsum and phosphogypsum were also reported in recent studies. They are advantageous over sulfur as they are slightly soluble in water, do not alter soil pH and can provide sulfate ions readily to plants [32]. Ibrahim et al. [33] varied gypsum and sulfur proportions as coating material and reported that an equal ratio of gypsum and sulfur results in the best efficiency and lowest urea release. Babadi et al. [34] also presented similar results with gypsum/ground magnesium lime coating. Efficiency was shown to improve by adding hydrophobic sealant such as paraffin and polyol. A urea release study from both studies showed similar urea concentrations of 2.15 mol/L and 2.5 mol/L, respectively, after 5 h. Despite having slower release properties than conventional urea, it is still faster compared to SCU. Phosphogypsum/paraffin CRF synthesized by Yu and Li [35] showed superior controlled release properties of urea compared to previous studies and satisfies the controlled release criteria by the European Standardization Committee (CEN) Task Force. This was due to the addition of emulsifier, Span− 80, which enhances the adhesion of the brittle paraffin coating. Its release rate also slows down significantly as the particle size and thickness of paraffin coating increase. 

Minerals such as hydroxyapatite, bentonite, zeolite and attapulgite were also explored as they can act as soil conditioners and improve the physical and chemical properties of the soil as well as ion-exchange properties that favor plant growth [36]. Kottegoda et al. [37] used nanotechnology to develop urea-hydroxyapatite (HA) nanohybrid CRF. Release studies in water showed that the Urea-HA nanohybrid CRF released urea slowly for up to a week. This was attributed to the moderately strong bond between the amine group of urea and carbonyl group of hydroxyapatites. It was also proven in field applications to be able to save up to 50% of urea consumption. Elhassani et al. [38] developed urea-impregnated hydroxyapatite encapsulated with lignocellulosic biomass, which further retards the initial release rate and sustains the release for up to 55 days to release 75% of the nutrients, compared to 3 days for an unmodified formulation, due to its hydrophobic nature. 

Dubey and Mailapalli [39] formulated zeolite coated urea fertilizer using different binders (corn and potato starch, bentonite, white cement, acrylic polymer). The acrylic polymer was shown to be the most effective binder as it forms a stable CRF with high crushing strength and slowly releases 54% of N after 8 h in water. Pereira et al. [40] prepared CRFs using bentonite nanocomposite modified with various concentrations of hydrophilic (polyacrylamide) or hydrophobic (polycaprolactone) polymer. Polyacrylamide hydrogel was more effective as it has good interaction with bentonite/urea nanocomposite, which competes with water and slows down urea release. Moreover, bentonite is electrically balanced by cations which allows NH_4_^+^ ions to be adsorbed to the matrix. The slowest cumulative release is 8% in 8 h [40]. This was superior compared to the urea, bentonite and organic polymer composite prepared by Xiaoyu et al. [3], where the slowest cumulative release is 45% in 8 h. Hermida and Agustian [21] formulated CRF by incorporating bentonite using starch and hydroxypropyl methylcellulose (HPMC) binders. CRF with HPMC binder has faster release due to its hydrophilicity, while the one with starch content released 25% urea in 8 h. This study suggested that these minerals and binders form physical attraction through Van der Waals forces, hydrogen bonds and electrostatic attractions between molecules, which aids in slowing down the release. Attapulgite modified with ethyl cellulose (EC) and carboxymethyl cellulose and hydroxymethyl cellulose (CMC/HEC) hydrogel was prepared by Ni et al. [41], and similar to bentonite, was able to retard release due to its adsorption capability. However, the release was much slower compared to previous studies (15% in 3 days) due to an optimum EC and CMC/HEC hydrogel ratio and optimum crosslinker content. Table 1 summarizes the inorganic materials used to formulate CRFs and the duration required to release 75% of their nutrient content. 

### 4.2. Synthetic Polymer-Based Coatings

Following sulfur and other inorganic materials, polymer coating materials become more favorable as they are insensitive to environmental factors and can be altered for the controlled release of fertilizers. Release patterns from polymeric coatings depend on their thickness and soil temperature, which will affect diffusion [42]. The use of single or blended polymeric materials was developed to solve the problem of quick and instantaneous nitrogen release. 

Yang et al. [43] prepared polystyrene with wax and polyurethane (PU) additive for coating urea. An experiment shows that PU is more effective in reducing release rate with the same coating percentage, as wax cannot prevent water from penetrating the coating at early stages of release. Increasing tablet size also reduces the rate and coating material required, hence lowering its production cost. Li et al. [44] developed coated urea using pure PU and mesoporous silica filler with different morphologies (fibrous, nanorod and spherical). Different morphologies significantly influence the pore structures, such that they may agglomerate or cause defects which affect the release rate. Rod-like morphology was found to be the most effective as it forms an interpenetrating network between PU and mesoporous silica and is able to release 80% of its content over a period of 80 days. Dai et al. [45] synthesized coated urea fertilizer with different levels of hydrophobicity by copolymerizing PU and hydroxypropyl-terminated polydimethylsiloxane (HP-PDMS). Implementing inner hydrophilic and outer hydrophobic gradient layers increases the diffusion resistance of urea and allows its release over more than 60 days. Gradient hydrophobic coating layers also reduce the coating thickness required to achieve the same release rate by uniform coating, which significantly reduces cost. 

A new class of polymer, polyether sulfone, was also used together with Fe_2_O_3_ nanofiller as a CRF [46]. The addition of Fe_2_O_3_ nanoparticles (NPs) thickens the coating layer, which slows down the release of nutrients. In addition, Fe_2_O_3_ NPs allow the capsules to be recovered using magnetic power and reused, although the release rate tends to increase after 2–3 cycles due to the accumulation of content from the previous application. Due to rising environmental concerns, biodegradable synthetic polymers as coating materials were actively explored. Synthetic biodegradable aliphatic polyesters are hydrophilic and susceptible to hydrolytic degradation. This was confirmed by Ye et al. [47] and Bi et al. [48] by using different kinds of aliphatic polyesters as the coating material. These studies reported that increasing the size of coated fertilizer while using smaller crystals of urea dispersed in the matrix slows down degradation and the release rate. Degradation up to 82% after 3 months was reported by Ye et al. [47]. Li et al. [49] also formulated bio-based epoxy coatings by using different liquified bagasse (LB) to bisphenol-A diglycidyl ether (BDE) ratios, which significantly affect the material property and release characteristics. Increasing BDE to an optimum amount increases the compactness and hydrophobicity, which retards the release rate. 

Furthermore, hydrogel is receiving much attention due to its ability to absorb a large amount of water to reduce irrigation frequency and improve water retention in soil. Urea release using polyvinyl alcohol-based hydrogel was synthesized by Sarkar and Sen [50]. It can swell up to 250% and release only 15–20% urea on the first day. It was also reported that this CRF could adsorb Fe (III) ions due to its affinity to urea and reduce its toxic effects to plants. Chen et al. [51] formulated PVA/biochar CRF since the results of hydrophilic PVA alone on release rate was limited. Biochar is used as a support material to enhance mechanical strength and improve biodegradability as it can adsorb microorganisms. Rice biochar was shown to be the most effective comparative to biochar from other botanic origins as it has less hydrophilic OH- groups. Urea was encapsulated more compactly and densely and released 60% of nutrients in 22 days. Table 2 summarizes the synthetic polymers used to formulate CRFs in recent years and the duration required to release 75% of their nutrient content.

### 4.3. Natural Polymer-Based Coatings

Although synthetic polymers can be tuned and modified to obtain desirable properties of CRFs, non-biodegradable polymers have a major negative impact on the environment. After releasing their nutrient contents, the remaining polymer materials will remain and accumulate up to 50 kg/ha per year in the soil, causing white pollution [52]. This drives the research interest to natural polymers that are biodegradable and non-toxic to the environment. Natural polymers are commonly used with other materials to form composites, as natural polymers alone do not have adequate mechanical integrity and other properties ideal for a CRF. 

Several papers have studied oil-based polymer coating material. For instance, Yang et al. [53] prepared a double-layer polymer coated urea (DPCU) CRF using corn stover-based polyurethane as the inner layer and superabsorbent from chicken feather meal as the outer layer. They reported that hydrogel does not have any effect on the diffusion of urea as it only improves water retention in soil. The release rate was thus controlled by the coating thickness of the inner layer. The DPCU showed great controlled release properties as 75% of the nutrient content was released in 35 days. Bortoletto-Santos et al. [54] studied soybean and castor oil-based polyurethane coatings. They reported that castor oil-based PU provides better adherence to urea surface, which results in prolonged release times. The 7.5% soybean and 5% castor-based coating PU released urea in 40 days, showing that the same performance can be achieved using lower coating thickness and castor-oil based PU. Modified alkyd resin prepared with castor oil or rubber oil blended with starch was prepared by Uzoh et al. [55], agreeing that castor oil can provide superior controlled release properties with lower cost. Bortoletto-Santos et al. [56] further studied that castor-oil based PU also reduces N_2_O emissions without impacting the yield of maize grain. However, this study was specific to sandy soil and the results might differ in different soil types. These results also agree with the study conducted by Liu et al. [57]. However, this research incorporated nano-fumed silica (thickening agent), which was found to reduce porosity and pore size while slowing down release rates. Dong Feng et al. [58] prepared soybean oil-based PU coated urea. The team reported that the isocyanate index used in this study affects the morphology, crosslinking density, water absorption and the release rate. Higher isocyanate index results in higher cross-linking degree and lower water absorption, which decreases concentration gradient and slows down the release. The degradation rate ranges from 10.23% to 29.63% after 180 days, which increases with increasing vegetable oil-based polyol content. Elastic biobased PU coating by modification of acrylonitrile prepared by Liu et al. [59] showed enhanced swelling capacity and a slow nutrient release of up to 80 days, as compared to 50 days by normal biobased PU. 

Liu et al. [60] synthesized polysulfone using SO_2_ and eugenol as a coating material and showed a superior nutrient release of 70% in 30 days. Increasing molecular weight (M_w_) of the polymer decreases the rate of degradation as the intrusion of moisture is prevented. Yang et al. [61] developed hydrophilic coating using latex, but in contrast to Yang et al. [53], it was reported that swelling degree is the main factor affecting the release rate. Urea content strongly affects the swelling degree as it forms H-bonds easily with water and hinders the H-bond formation with polymer chains, which inhibits the diffusion of the water molecule. Riyajan et al. [62] prepared coating materials using natural rubber grafted with cassava starch (NR-g-ST), which is robust, rigid, and hard to swell. Increasing starch content reduces the hydrophobicity of natural rubber (NR) and improves swelling. NR-g-ST forms a dense structure which reduces diffusion rate through swollen beads (21% in 24 h). Cui et al. [63] agree that NR plays a vital role to retard the release rate due to its hydrophobic nature. They prepared a double-coated CRF by entrapping urea in attapulgite matrix, coated with NR as the inner layer and NR-g-polyacrylamide (NR-g-PAA) as the outer layer to enhance hydrophilicity. This multicoated hydrogel provides great controlled release through delayed swollen beads of the hydrogel (44.37% in 30 days). 

Starch is the most researched natural polymer for coating material due to its availability and low cost. Rychter et al. [64] prepared a starch based CRF with urea acting as the plasticizer. It was shown that plasticizer reduces moisture content, which affects the mechanical properties and crystallinity of the matrix. Higher urea content slows down the release, but it was reported that it was not satisfactory (75% nutrient release in 12 h) and further modification must be made to increase hydrophobicity for long term applications. This agrees with the work by Niu and Li [65], who used starch grafted with vinyl acetate which increases the hydrophobicity. This reduces swelling abilities, increases encapsulation efficiency and decreases nutrient cumulative release to 50% in 30 h, which is slower than the previous study. Giroto et al. [66] developed a starch/melamine/urea CRF and reported that higher melamine content slows down the release due to interaction between the amine group of urea with melamine and starch (40% in 120 h). Versino, et al. [4] reported similar trends as the team developed starch-based coating material with urea as the plasticizer, but with bagasse as a reinforcing agent. This improves the mechanical properties and, coherent with previous studies, increasing urea content and reinforcing agent promotes interactions which retard release. This composite provides better slow release compared to previous studies by releasing 95% of urea in 15 days.

Many starch-based hydrogel CRFs were developed in recent years due to the benefits associated with water retention, as mentioned previously. Jin et al. [67] developed starch/poly(acrylic acid-co-acrylamide) superabsorbent (SAAmF), which is partially degradable. Increasing the starch to poly(acrylic acid-co-acrylamide) ratio decreases water absorbency because the aperture in the 3D network reduces. This results in a slower release of 55% in 30 days. This was agreed by Xiao et al. [68] and Wen et al. [69] with similar cumulative release. However, in a study by Wen et al. [69], bentonite was incorporated in the hydrogel, which improves adsorption and complicates the path of nutrients, which contributes to slower release. Salimi et al. [70] synthesized starch hydrogel reinforced with natural char nanoparticles (NCNP). The release rate was reduced with increasing NCNPs because of favorable interactions between interfacial polymer and fillers. It was reported that 70% of the nutrient was released in 21 days. Qiao et al. [71] prepared a double-coated CRF with EC as inner coating and starch-based superabsorbent as an outer coating material. Using potato starch had the most significant impact on reducing grid size and increasing fractal gel size, which contributed to increased water absorption with slower absorbing rate. Hence, starch from different botanical origins greatly affects the morphology. Together with hydrophobic EC, the formulation improved release rate, although it did not fulfill the European Committee for Standardization (CEN EN 13266). Patil et al. [72] also performed similar work using cellulose nanofibrils (CNF) from waste sugarcane bagasse as filler. Although the reported release rate was not satisfactory, characterization showed that this formulation had 3.5 times lower surface area compared to neat urea and had a higher overall release of the active compound. This creates a great potential for making cost-effective CRF as the content is utilized to its fullest. All works with starch-based hydrogels show superior water holding capacity and retention in the soil compared to those without hydrogel CRF.

Cellulose and lignin are attractive raw materials for coating as they are the most abundant natural resources with low cost. Xie et al. [73] developed a novel macromolecular fertilizer poly(dimethylurea phosphate) (PDPU) which has a lower solubility than urea. It was proven that PDPU alone acts as a physical barrier and slows down the release. However, using wheat straw superabsorbent coating further enhances the performance as it can slowly swell to become a hydrogel and slowly release 67.6% of the nutrients in 30 days. Li et al. [74] also performed similar works but the release was reported to be 85% in 8 days, which was likely to be caused by fast urea dissolution compared to PDPU. Bortolin et al. [75] developed hydrogel composed of polyacrylamide (PAAm), methyl cellulose (MC) and montmorillonite (MMT) NPs. MMT decreases water absorption and, agreeing with the previous studies, it favors adsorption in the nanocomposite, which increases loading. Moreover, hydrolysis treatment of the hydrogel decreases pore size with increasing clay content and increases the amount of urea desorbed in a longer period, maximizing its utilization. Work by Wen et al. [76] also shows that incorporating bentonite increases porosity and compactness, which creates a tortuous path and retards diffusion (60% release in 30 days). Olad et al. [77] formulated a carboxymethyl cellulose-based nanocomposite with silica NPs, which increases water absorption and reduces the cost of formulation. A total of 56.4% of the fertilizer was released in 30 days. Mulder et al. [78] worked with different commercial lignins as the coating material. Soda flax lignin (Bioplast) with the addition of hydrophobic alkenyl succinic anhydride (ASA) and crosslinkers shows the greatest potential for slow release due to its water repelling properties, although the release rate is still too high for industrial applications. 

Alginate is a natural and biodegradable polymer extracted from marine algae, reported by various studies to be the most non-toxic, which readily forms beads by crosslinking in the presence of divalent cations, such as CaCl_2_ [79]. Rigid shells are then formed where the substances are trapped in the core. Wang et al. [15] developed a double-coated CRF with the inner layer coated with sodium alginate (NaAlg)/κ-carrageenan (κC) and the outer layer coated with κC grafted with celite superabsorbent (κC-g-PAA/celite). This formulation achieved 343% of the swelling and a slow release of 90% in 25 days through optimum content of crosslinker, κC and celite, which affects the morphology. Rashidzadeh and Olad [80] also formulated alginate-based superabsorbent incorporated with MMT to form a nanocomposite. This creates a highly porous structure, which increases water absorption. The formation of a tortuous structure retards diffusion and induces a slow release of 68.34% of nutrients in 30 days. The incorporation of biogenic silica in the alginate matrix by De Matos et al. [81] also reported similar effects and had a cumulative release of 85% in 60 days. The incorporation of CNF into alginate matrix was also investigated; however, it agrees with the previous findings from Patil et al. [72] that it has no effect on release properties, but serves as a potential raw material for more sustainable preparation. 

Chitosan is a polysaccharide derived from the exoskeleton of crustaceans and cell walls of fungi, which exhibits antifungal and antiviral properties in plants [20]. Several works using chitosan have been carried out. Araújo et al. [82] formulated chitosan-based coating material using humic substances (peat, humic acid, humin). It was reported that, depending on the type of humic substances and the pH of the aqueous medium, it will affect the release rate differently due to the functional group and possible interactions of each compound with urea. Work by Huey et al. [83] involved the use of allicin, which is a urease inhibitor in chitosan/starch composite. It was reported that allicin lowers the hydrolysis rate of urea and postpones the availability of nutrients to plants. Adlim et al. [84] developed urea-magnesium-natural rubber composite coated with chitosan. They reported that magnesium interacts with urea to become solid and become trapped in the rubber matrix, holding urea release. However, it was mentioned that chitosan does not provide any significant effect on the release. Iftime et al. [20] formulated chitosan hydrogel with salicylaldehyde, which can act as a soil conditioner due to larger water holding capacity in soil and a reduced water evaporation rate. Urea is anchored by H-bonds into the chitosan hydrogel mostly as submicrometric crystals. So, larger urea crystals will be less anchored into the matrix and result in faster release. Table 3 summarizes the natural polymers used to formulate CRFs and the duration required to release 75% of their nutrient content.

### 4.4. Other Organic Material Coatings

Several other organic materials that were not under the category of polymers were discussed. Their application may also promote chemical and biological properties of the soil and ion exchange [85].

Wen et al. [86] prepared a CRF with biochar/super absorbent polymer grafted with bentonite to increase its water retention properties. Biochar was reported to have a strong sorption capability, which makes suitable for CRF application. A slow release of 70% of the content in more than a month was reported. Similar theory and cumulative release were reported by Shi et al. [31]. It was also reported that the proper mineral binder could enhance N retention through surface adsorption and organic/mineral interaction. Mumtaz et al. [29] prepared a coated CRF using rosin adduct with maleic anhydride, and it was reported to be an effective barrier to slow down urea release (45% in 14 days). This can be due to the covalent bonds between the carbon in maleic anhydride and nitrogen in urea. It was also shown to work effectively under different soil textures. Table 4 summarizes the organic materials used to formulate CRFs in recent studies and the duration required to release 75% of their nutrient content.

According to the criteria of CRF as recommended by the European Standardization Committee (CEN) Task Force, no more than 75% of the nutrients should be released within 28 days [23]. Based on the summary of the time frame of release in Table 1, sulfur, mineral and inorganic-based CRFs did not fulfill the criteria as the nutrients were quickly released in a matter of minutes, hours or a few days. This was due to the brittle nature of the main materials, which makes the coating susceptible to cracks. However, the use of certain fillers which have hydrophobic properties (Span− 80) and good adsorption capability (bentonite, sepiolite) helped to prolong the release rate.

On the other hand, for synthetic and natural based polymer CRFs, the formulations that fulfilled the release criteria are those that had certain degrees of hydrophobicity such as polyurethane, natural rubber, or the presence of hydrophobic gradients in the coating. Hydrophobic fillers (polyurethane) and fillers with good adsorption capability (bentonite, montmorillonite, natural char, biochar) play major roles in determining the release rates of fertilizer. The ideal properties of the CRF must be sufficiently hydrophilic and hydrophobic, and the nutrients must be able to adsorb well on the CRF matrix for optimum performance.

## 5. Coating Techniques

The coating methods of controlled release fertilizers can be divided into physical or chemical processes. Physical methods such as spray coating using a rotary drum, pan coater and fluidized bed technologies are well known, well developed and have been implemented commercially even until today. These spray coating techniques are a continuous process with low operating costs. They are also easily scalable, which makes them attractive for industrial-scale processes. However, several drawbacks come with these techniques. 

Sprayed coatings form more porous membranes than casted ones, which makes their structure difficult to predict unless they undergo experimentation [87]. Coating using a rotary drum requires a large volume of raw materials, as a lot of it will be wasted to achieve uniform coating [88]. This increases the cost of raw materials. This method was reported in various studies that use polyurethane, epoxy, and gypsum as the coating materials [33,49,51,53,58,59,60]. The rotary drum is usually pre heated to around 70 ± 2 °C for 5 to 10 min before placing the urea prills. The coating is carried out using side spray nozzles at 0.7 MPa and the drum is rotated at 45° and spun at 60 rpm. The process of preheating, coating, cooling, and collection can take up to 1 to 3 h. 

On the other hand, in pan coating, a coating solution is sprayed to urea granules under air high temperature for drying purposes [88]. This often results in poor coating quality (defective porous layer) due to poor maintenance of the humidity level during production [89]. It was also reported that coating uniformity using this method is affected by particle size distribution, binder properties, the number of coating materials and drying temperature [27]. This method is reported in various studies that use clay minerals and natural as well as synthetic polymers [15,35,39,54,56,63,71,78]. Granules with the desired size range are obtained under water atomization and coating solution is sprayed while the pan is tilted to 45° and rotating at 16 to 30 rpm. Dubey and Mailapalli [39] reported that at least 20 min was required to achieve uniform coating thickness. Then, coated granules are dried using hot air of 130 to 140 °C to remove excess water. 

Fluidized bed spray technology yields more uniform coatings by using melt or liquid coating materials. Moreover, it also allows a wider selection of coating materials, either non-solvent mediated, solvent-mediated, hydrophilic or hydrophobic [90]. The process is controlled by adjusting many variables, such as spraying rate, coating cycle and temperature [30]. However, some drawbacks include expensive equipment, long residence time, being prone to filter blockage, higher chances of solvent explosion and poor performance with granules of a larger size as they affect trajectory [91]. This method is reported in various studies that use synthetic and natural polymers [30,43,45,61]. The fluidization bed is pre-heated with fluidization gas to 45–50 °C for 5 min. In a study by Dai et al. [45], the air was compressed to 80 °C. The coating solution was pumped into the nozzle and atomized at a set pressure and set flow rate depending on the process. Yang et al. [43] used a pressure of 0.3 MPa while Wang et al. [30] used pressures between 1.8 and 2 MPa. The granules were then dried at 54 to 60 °C in the fluidized bed. 

However, these spray coating techniques often require the use of organic solvents to dissolve the resin and control coating evaporation rates and viscosity, which affects coating adhesion and durability [92]. The evaporation of organic solvent poses a hazard to human health and the environment [93]. Thus, environmentally benign techniques such as melting and extrusion using single or twin-screw extruders were developed to overcome this problem. This method is also facile, cheap and does not involve high pressures [48]. However, hot melts are involved in the process and the equipment is expensive [94]. Several works using starch, polyesters and clay minerals use this method to prepare CRF [3,21,38,40,48,64,66,68]. Torque and temperature must be monitored closely when performing melting and extrusion techniques. Rychter et al. [64] reported CRF preparation using a single screw extruder provided with six different heating zones, where the screw rotation was operated at 50–60 rpm and with torque and melt pressure of 80–100 Nm and 25–30 Ba, respectively.

Chemical processes are often used to prepare hydrogel CRFs using superabsorbent polymers. The preparation method includes solution polymerization, inverse suspension polymerization and polymerization by irradiation. Monomers, initiators and crosslinkers are the most important factors in hydrogel preparation as the concentration affects hydrogel properties [95]. Solution polymerization, also known as a cross-linking reaction, is carried out by mixing the monomer and initiator, which must be soluble in the chosen solvent. The solvent used reduces the viscosity of the reaction which eases the operation. However, it is hard to recover the solvent since monomer and initiator are mixed with multifunctional crosslinking agents and the slower rate of reaction results in lower encapsulation efficiency. Inverse suspension polymerization uses hydrophilic monomers and initiators dispersed homogeneously in hydrocarbon phase (water in oil) and needs to be constantly agitated since it is thermodynamically unstable [95]. Since the resulting reaction is insoluble in the solvent, the solvent can be recovered, which can save costs, and it has a higher rate of reaction which may improve encapsulation efficiency. However, there may be possible contamination within the suspension, which requires further purification processes downstream. 

These methods are used to produce hydrogel CRFs using natural polysaccharides including starch [67,72], alginate [80] and gelatin [96], or synthetic hydrophilic polymers such as polyvinylpyrrolidone [77] grafted with acrylic acid and acrylamide monomer. *N*,*N*-methylene biacrylamide (MBA) as cross-linker and ammonium persulfate (APS) as initiator are also the most commonly used. Most of the formulations are soluble in water, making the process environmentally benign. However, some formulations require the use of organic solvents such as isopropanol, *N*,*N*-dimethylformamide (DMF) and 2,2-dimethoxypropane (DMP). De Matos et al. [81] prepared a sodium alginate-based hydrogel CRF by cross-linking with CaCl_2_.

Another method of polymerization is by irradiation, which results in the formation of macroradicals. When these macroradicals are recombined on different chains, it results in covalent bonds and cross-linked structures [95]. Cotton stalk, corn cobs and biochar based semi-interpenetrating networks (IPN) were synthesized using microwave irradiation at 320 W for 4.5 min as a greener pathway of chemical synthesis due to simplicity, high efficiency, and low energy consumption [69,76,86]. However, this method is yet to be widely implemented in the preparation of CRFs. The coating techniques are summarized in Table 5.

## 6. Important Factors Affecting CRFs

As discussed previously in the materials section, the release rate of CRFs is generally affected by the size, coating thickness and uniformity, the selection of materials as well as the selection of binder and filler for the formulation. For hydrogels, the temperature, pH, and ionic strength of the environment also affect the nutrient release rate. 

### 6.1. Temperature

An increase in temperature reduces the duration of lag period and increases the linear rate of release [97]. Emami et al. [46] explained that as the temperature in the environment (soil) increases, the solubility of nutrients within the polymer and diffusion rate also increase, as diffusion coefficient is a function of temperature. In addition, pore size also increases with increasing temperature due to higher swelling, which results in higher release rates. It was also mentioned that as temperature increases by 15 °C, the release rate doubles. Bi et al. [48] reported that more rapid diffusion occurs at a temperature of 37 °C compared to 25 °C. They highlighted that the difference in temperature affects the degradation behavior in enzymatic environments. Similar findings were also reported by Uzoh et al. [55]. The temperature dependence of the linear release rate is represented by Equation (1):

(1) $$R_{\mathrm{lin}}=C^{0}{}_{\mathrm{sat}}\times P^{0}{}_{s}\exp\left(-\frac{{\mathrm{EA}}_{\mathrm{ps}}+{\mathrm{EA}}_{c}}{\mathrm{RT}}\right)$$

where R_lin_ is the linear release rate, $C^{0}{}_{\mathrm{sat}}$ is the reference standard values of solubility (g cm^−3^), $P^{0}{}_{s}$ is the reference standard values of permeability (cm^2^ d^−1^), EA_c_ is the activation energy associated with the solubilization of the fertilizer (kJ mol^−1^), EA_ps_ is the activation energy of its permeation through the membrane (kJ mol^−1^), R is the universal gas constant (kJ mol^−1^K^−1^) and T is the temperature (K). The overall activation energy of the release, ${\mathrm{EA}}_{\mathrm{ps}}={\mathrm{EA}}_{\mathrm{ps}}+{\mathrm{EA}}_{c}$, is an important parameter which demonstrates the sensitivity of nutrient release rate to temperature, and can be calculated by plotting R_lin_ against 1/RT [97]. The parameters of the CRF can be modified to alter the EA_rel_.

### 6.2. pH

**Figure 3:**
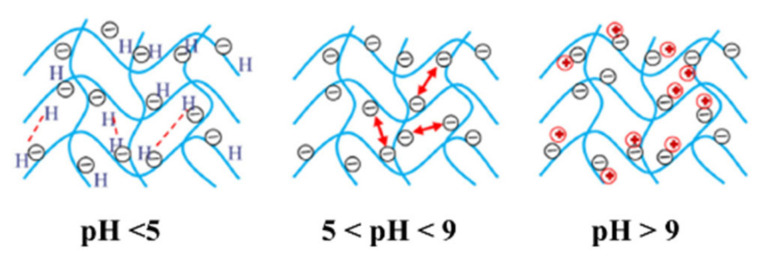
Schematic of the behavior of hydrogel in different pH. Reprinted with permission from [80]. Copyright 2014 Elsevier.

The acidic or alkaline nature of the release medium has a significant effect on the interactions of chemical species in the granule and the diffusion coefficient of the ions [98]. Rashidzadeh and Olad [80], Emami et al. [46], Wen et al. [69], Uzoh et al. [55] and Salimi et al. [70] reported that at an acidic environment (pH 2–5), there is a high concentration of H^+^ ions. This causes most of the carboxylate anions (COO^−^) to be protonated and prevents anion-anion electrostatic repulsion in the network, decreasing the swelling capacity. Similarly, at an alkaline environment (>pH 9), the presence of Na^+^ ions in the solution shields the COO^−^ anion and prevents anion-anion electrostatic repulsion. Between pH 5–9, or in a more neutral condition, the swelling capacity was expected to be the highest as the COOH groups are converted to COO^−^ ions, which maximizes electrostatic repulsion. Olad et al. [77] added that hydrogels are smart materials and respond well to pH as this work shows on-off swelling behaviors between pH 8 and pH 2, where slower release is observed in lower pHs with decreased swelling. The general behavior of hydrogel in different pHs is shown in Figure 3.

### 6.3. Ionic Strength

From the studies mentioned in the previous section, swelling capacity in a salt solution (containing NaCl, KCl, CaCl_2_, FeCl_3_) was also shown to be significantly lower compared to distilled water. This is attributed to the difference in osmotic pressure, which decreases due to the charge screening effect of the cations which shields the COO^−^ anions and reduces the repulsive force. The swelling capacity decreases in the order of Na^+^ > K^+^ > Ca^2+^ > Fe^3+^ [69,70,80]. With increasing charge (multivalent cations), it will form complexes with the carboxylate groups, which results in cross-linking points. This avoids the expansion of the hydrogel network, reducing swelling and the release rate. 

### 6.4. Granule Radius and Coating Thickness

Shaviv et al. [99] presented a mathematical model to predict the three different stages of release. It was reported that the product of the radius and coating thickness is proportional to the lag period, while it is inversely proportional to the release rate in the linear and decay phase. The study suggested that by either increasing the radius or coating thickness, the lag period can be prolonged, and the release rate can be slowed down in both the linear and decay phases. Increasing the radius of the CRF is generally preferred in the interest of economic feasibility. However, there is an always an optimum granule size required for the proper distribution of nutrients in the root zone. 

## 7. Mechanism of Release

**Figure 4:**
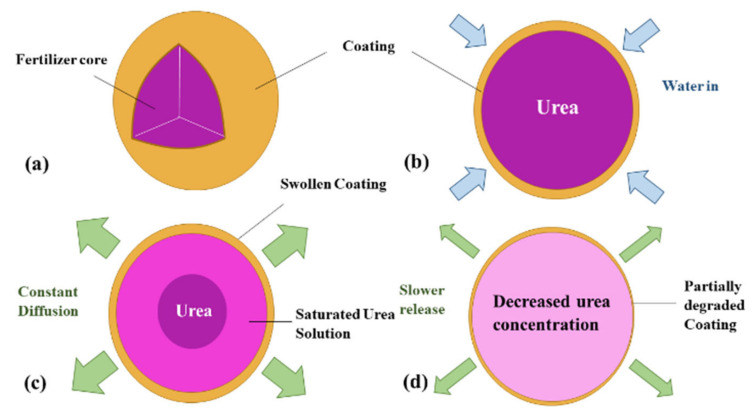
Mechanism of controlled release. (**a**) CRF granule. (**b**) A lag period where water penetrates through the coating to the core. (**c**) The buildup of internal pressure results in constant release to the environment. (**d**) Decay stage where concentration gradient and release rate decrease.

**Figure 5:**
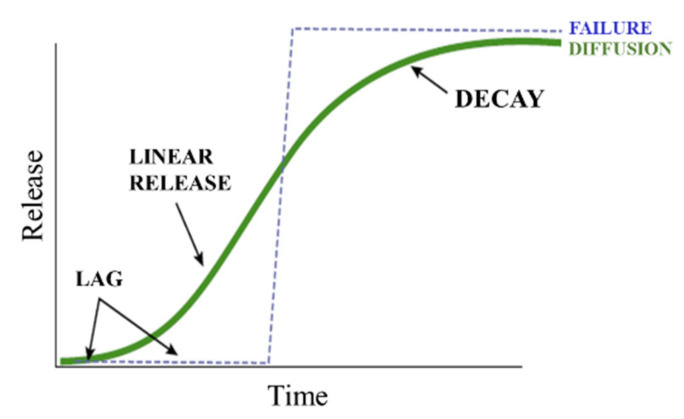
Sigmoidal release pattern (green) and failure release pattern (blue). Adapted from [23].

The release of nutrients from CRF generally takes place in three different stages: lag period, constant release and decay period [27,99]. In the first stage, water in soil, mostly in the form of vapor, wets the cracks present on the coating and penetrates to the core, where a small fraction of the urea fertilizer is dissolved. The vapor pressure gradient is the driving force and no fertilizer is released at this stage. In the case of hydrogel CRFs, they will absorb the water and swell. The lag may be due to the time needed to fill the internal voids with the critical water volume or to establish a steady state between the flux of water entering and flux of solute leaving [99]. In the second stage, as water keeps penetrating in, more solid fertilizer is dissolved and the osmotic pressure in the core builds up and the critical water volume of saturated solution accumulates, which allows the fertilizer to be slowly released through the cracks in polymer coating or the swollen hydrogel network. Since the concentration of the solution inside the granule remains saturated, the diffusion to the soil is constant [99,100]. If the pressure exceeds a prescribed threshold value, it results in the rupture of coating material and immediate burst release of the fertilizer content. In the decay stage, most of the fertilizer has been dissolved and released, which reduces the concentration gradient and the driving force and thus release rate. The mechanism described above is illustrated in Figure 4**.** It can also be described by a sigmoidal (S-shaped) release profile, as shown in Figure 5. This indicates that the release process is complex and non-linear. The sigmoidal release profile is what researchers aim to achieve through the formulations, as it shows controlled release characteristics matching the nutrient requirements of plants [99]. 

## 8. Predicting Nutrient Release of CRFs with Modelling

The release rate from CRFs is controlled by diffusion, swelling, erosion or a combination of these. Typically, when hydrophilic materials are involved, the release easily occurs through diffusion, whereas in hydrophobic materials, the release is associated with the swelling or erosion of the matrix [101]. Therefore, is it important to select the appropriate modelling approach for each formulation to obtain realistic theorical assumptions and understand the mass transport mechanisms involved to be able to come up with optimal CRF designs [100]. The modelling approaches could be categorized into mechanistic and empirical forms.

### 8.1. Mechanistic Approaches

The early mathematical model was developed by Jarrell and Boersma [102] for sulfur coated urea (SCU) by assuming that it occurs in a 1-D system, ignoring the effect of radius, and the model did not account for the lag period which was observed in many coated CRFs. Due to these limitations, they further proposed an expression for diffusion coefficient (D) which takes into account the dependence of release on temperature, In contrast, Glasser et al. [103] proposed a time-dependent expression for D which considers the lag period that cannot be predicted using Fick’s law.

Mathematical models for polymer-coated CRFs were first developed by Lu and Lee [104] for urea release in latex coated urea by applying Fick’s Law in spherical coordinates. They divided the process of release mechanism into two phases, namely linear and decay, but did not consider the lag period. They further proposed a model by taking into account population effects of granules based on pseudo steady-state mass balance equations of Fick’s law, and neglected the first stage of release and determined D by trial and error [105]. 

However, all these models were developed on the assumption that nutrient release is based on simple solute diffusion [106]. Shaviv et al. [99] used a mathematical model to predict the release in three different stages from a single granule by considering the measurable geometrical and chemo physical properties, such as granule radius, coating thickness, water and solute permeability, saturation concentration of fertilizer and its density. The equations for the fraction of release in three different stages are presented as follows (2):

(2) $$g(r,l,t)=\left\{\begin{array}{c} 0,\;t<t\prime \\ \frac{3P_{s}C_{sat}}{rl}\left(t-t\prime \right),\;t\prime \leq t<t*\\ \left(1-\frac{C_{sat}}{P_{s}}\right)exp\left[-\frac{3P_{s}}{rl}\left(t-t*\right)\right],\;t\geq t* \end{array}\right.$$

where g is the fraction released, r is radius, l is coating thickness (cm), *t* is time (day), *t′* is time at the end of lag period (day), t* is duration of the linear release period (day), $P_{s}$ is the solute permeability (cm^2^ d^−1^), and $C_{sat}$ is the saturation concentration (g cm^−3^).

Shen et al. [107] also predicted nutrient release from polymer coated urea (PCU) by considering the irreversible thermodynamics theory, where nutrient release is a function of the swollen radius of granules. The model was shown to be acceptable for the release profile of a single granule with a r^2^ = 0.864. However, the model was poor for the release profile from a population of granules under static conditions due to the reduction in driving force, requiring further research. This could be explained by the fact that when a population of granules was considered, large variations in the properties are expected due to coating defects, which in turn make the release characteristics significantly different from a single granule [108]. It was found that in the presence of coating defects, the solute permeability is inversely dependent on the coating thickness, $P_{s}=P_{s}/l$. This resulted in non-linearity of the release pattern and equations (2) were then modified as follows (3) [108]:

(3) $$g(r,l,t)=\left\{\begin{array}{c} 0,\;t<t\prime \\ \frac{3P_{s}C_{sat}}{rl^{2}}\left(t-t\prime \right),\;t\prime \leq t<t*\\ \left(1-\frac{C_{sat}}{P_{s}}\right)exp\left[-\frac{3P_{s}}{rl^{2}}\left(t-t*\right)\right],\;t\geq t* \end{array}\right.$$

The finite element method (FEM) has been used to study the release of nutrients from CRFs since 2003 [99]. As urea release is a complex process, Trinh et al. [106] further studied the constant release stage of urea in water by using mass transport equations in a porous medium (4). The flux of urea from the interface to the liquid was assumed to be controlled by the diffusion of urea in liquid as the pellet is motionless, which simplifies the equation to (5):

(4) $$D_{e}\left[\frac{\partial^{2}C}{\partial r^{2}}+\frac{2}{r}\frac{\partial C}{\partial r}\right]=\xi \frac{\partial C}{\partial t}$$

where $D_{e}$ is effective diffusivity (cm^2^ s^−1^) and ξ represents porosity, C is the concentration (g cm^−3^), r is the radius of coated urea (cm) and t is time (day).

(5) $$D_{\mathrm{urea}}=\left(1.380\;-\;0.0782C\;+\;0{.00464C}^{2}\right)10^{-5}{\;\mathrm{cm}}^{2}/s$$

where $D_{\mathrm{urea}}$ is the diffusivity of urea in liquid (cm^2^ s^−1^) and C is the concentration (g cm^−3^). 

However, these models only cater to the first and second release stages without considering the decay release stage. Trinh et al. [109] further developed this work by applying multidiffusion mechanisms to obtain a sigmoidal release profile which includes the third stage of release (6):

(6) $$C_{\mathrm{DR}}\left(R_{0},t\right)\;=\;\frac{m_{\mathrm{core}}(t)\;}{V_{\mathrm{core}}}\;\mathrm{at}\;t\;\geq {\;t}_{1}$$

where $C_{\mathrm{DR}}\left(R_{0},t\right)$ is the decay concentration at the surface of urea core after the end of constant release at any time (g cm^−3^), $m_{\mathrm{core}}$ is mass of urea core (g) and $V_{\mathrm{core}}$ is the volume of urea core (cm^3^). 

**Figure 6:**
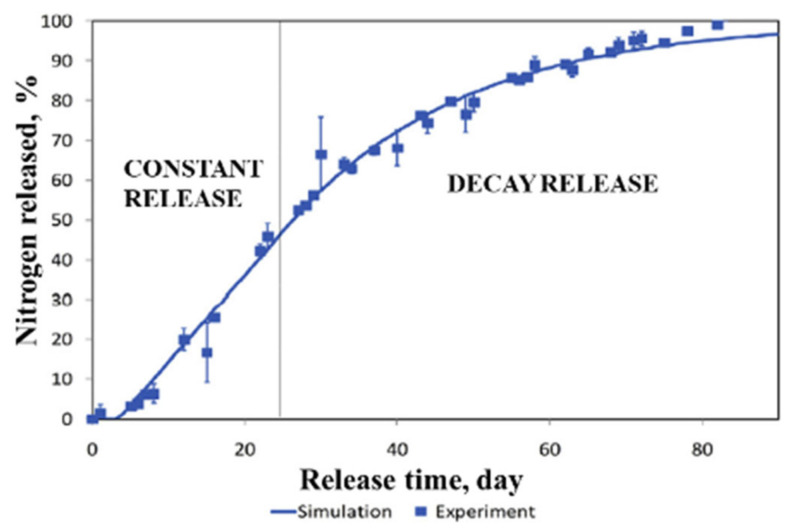
Release profile of Agrium. Reprinted with permission from [109]. Copyright 2015 Elsevier.

**Figure 7:**
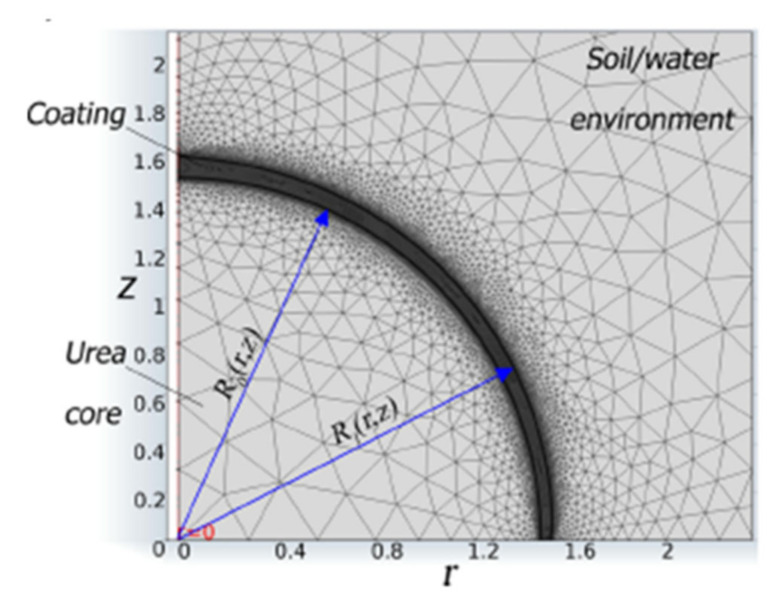
Geometry and Mesh Generation of urea core and its environment. Reprinted with permission from [110]. Copyright 2015 American Chemical Society.

The simulation results were validated by comparing them with the release test results of agrium, which corresponded well with each other with a standard error of estimate of 0.03. Both of the release profiles are shown in Figure 6. This model can be used to predict the release of a wide range of PCUs in water. As the nutrient release profile of PCU in field conditions is more relevant and can be used to support existing research, Trinh et al. [110] expanded the model to account for the effect of coating imperfections and different soil charactertistics using (7). Assumptions of no soil water movement, constant temperature during release and no loss of N to the environment were also made. Figure 7 shows the geometry and mesh generation of a urea granule and its environment in 2-D coordinates.

(7) $$\xi \frac{\partial C_{i}\;}{\partial t}{\;+\;C}_{i}\frac{\partial \xi \;}{\partial t}\;+\;\nabla \left(C_{i}u\right)\;=\;\nabla \cdot \left({\theta \tau }_{F,i}D_{F,i}\nabla C_{i}\right)$$

where ξ is porosity, $C_{i}$ is the concentration of species in liquid (g cm^−3^), u is the velocity of soil water movement (ms^−1^), θ is the water volume fraction, $\tau_{F,i}$ is the dimensionless tortuosity factor, $D_{F,i}$ is the diffusion coefficient of i residues in pure water at infinite dilution (cm^2^ day^−1^) and t is time (day).

### 8.2. Empirical and Semi-Empirical Approaches

Mechanistic models are mostly developed based on the assumption that nutrient release is mainly governed by diffusion, which may not be the case for all CRFs. For this reason, empirical and semi-empirical models are often used to aid researchers to obtain an idea of the relationship between variables that represents a particular system to understand the governing mechanism. The Generalized Neural Network Model (GRNN) is an empirical model used to predict the nitrate release profile [111]. The Higuchi model and the Ritger–Peppas and Korsmeyer–Peppas models (Power Law) are also common empirical and semi-empirical models, respectively. 

#### 8.2.1. General Neural Network Model (GRNN)

Du et al. [111] used the GRNN network empirical model to predict the nitrate release pattern from polymer coated fertilizer (PCF). GRNN is a radial basis neural network that can approximate any arbitrary function between input and output vectors, drawing the function estimate directly from the training data. This model was validated as the results are close to the results obtained experimentally. It was also reported that GRNN was a simpler and more accurate approach to predict the nutrient release as compared to the theoretical model. Furthermore, it can be used to design the PCF by optimizing the parameters to obtain the desired release rate. 

#### 8.2.2. Higuchi Model

This empirical model is used to describe the release of soluble or less soluble nutrients from a matrix system. Some assumptions need to be made when using the Higuchi model (8): the initial concentration of nutrients in a matrix must be higher than their solubility, diffusion occurs in one direction (edge effects are negligible), negligible swelling, nutrient particles are much smaller than the thickness of the coating, constant diffusivity, and, lastly, perfect sink condition is achieved in the release environment. Higuchi models are used to develop a variety of other mathematical models, including Peppas models [101]:

(8) $$Q=\sqrt{{2C}_{0}\xi \frac{\mathrm{Dt}}{\tau \pi }}{=K}_{H}\sqrt{t}$$

where Q is the cumulative amount of fertilizer released at time t per unit area (g cm^−2^), $C_{0}$ is the concentration of diffusing liquid contained in a porous matrix (g cm^−3^), ξ is porosity, D is the diffusion coefficient in the matrix medium (cm^2^ day^−1^), τ is the capillary tortuosity factor, t is time (day) and $K_{H}$ is the release constant of Higuchi.

#### 8.2.3. Ritger–Peppas and Korsmeyer–Peppas Models (Power Law)

This semi-empirical model specifically describes the release of materials from the polymeric matrix using an exponential relationship between the release and time described by (9) [101]. This model would be useful when the release mechanism is not known or when more than one mechanism is involved.

(9) $$f_{1}=\frac{M_{i}\;}{M_{\infty }}{=\mathrm{Kt}}^{n}$$

where $f_{1}$ is the fraction of nutrient released, $M_{i}$ is nutrient released over time t (g), $M_{\infty }$ is the total amount of nutrient (g), K is the release velocity constant, t is time (day) and n is the exponent of release.

The release mechanism could be classified into a Fickian model (Case I) or Non-Fickian models (Case II, Anomalous Case and Super Case II) depending on the value of n that suits the release profile [101]. When n = 0.5, the model is Fickian (Case I) and the release is governed by diffusion, which is much greater than polymeric chain relaxation. When n = 1, the model is non-Fickian (Case II) and approaches zero-order kinetics, where the release is governed by swelling or relaxation of polymeric chains. When 0.5 < n < 1, the model is non-Fickian (Anomalous Case) and the release rate is governed by both diffusion and swelling, while n > 1 represents an extreme case (Super Case II model) where polymer chains break during the sorption process.

The modification of materials to increase hydrophobicity and increase coating thickness results in zero order or sigmoidal release profiles which follow non-Fickian models (Anomalous Case), which are more desirable [21,54,59]. Using a smart hydrogel CRF that induces change of properties with pH also affects nutrient release, whereas the use of chitosan with peat and humin results in an anomalous or super case II model depending on the pH medium [82].

## 9. Commercial Uses

There are many patents located by Scopus that were filed on the invention of controlled release fertilizer (CRF). They mainly focused on creating better formulations to improve physical properties and increase the utilization rate of fertilizer nutrients. Recent patents showed that the majority of the inventions employed biodegradable coating materials such as polylactic acid (PLA), okara (soy pulp), linseed, polyurea and corn starch hydrogel [112,113,114,115,116], while some involved the use of polyurethane and resin [117,118]. Fillers ranging from silicate, gypsum, corn starch, microcrystalline cellulose, bentonite, and other bio-based additives are often incorporated in the formulations. These CRFs can be produced using different encapsulation techniques such as spray coating, drum coating and fluidized bed. The reported applications of CRF focus mainly on plant growth promotion of field crops such as wheat, rice, corn and cotton, and vegetables such as choy sum as well as ornamental plants. CRFs possess slow nutrient release patterns that match the needs of these crops. The patent findings demonstrate the commercial potential of CRFs as an alternative to existing agricultural fertilizers.

## 10. Conclusions and Future Outlook

There is a growing number of researchers utilizing natural polymers for the formulation of CRFs. Binders and fillers play a vital and important role in the release pattern as they can form compact structures, altering the properties of pore size and interacting with urea, which favors adsorption. The properties of the materials that make CRFs sufficiently hydrophobic are essential for achieving controlled release without immediate disruption of the coating wall. CRFs can be prepared using physical and chemical methods. Methods of polymerization by microwave irradiation are proven to reduce energy consumption. The temperature, pH, and ionic strength of the environment significantly govern the nutrient release behavior. Mechanistic, empirical, and semi-empirical approaches have been widely used for modelling nutrient release from CRFs. Nevertheless, it should be mentioned that CRF is a broad field of study that is constantly changing and evolving, with multiple aspects yet to be examined and reviewed. Thus, the focus of future works can be narrowed down to focus on the utilization and formulation of low-cost biodegradable materials as a blend or with suitable binders that favor adsorption and provide sufficient hydrophobicity. Field testing under different environmental conditions is needed to validate the results and to study the tailing effect of release towards the end-of-life of CRFs. The formulation of procedures and methods for upscaling should also be developed with the goal of bring CRFs to a point of practical and commercial application.

## Figures and Tables

**Table 1:** Sulfur and mineral-based CRFs.

Sulfur-Based				
Material	Modifier	Research Findings	Release Duration ^a^	References
Gypsum	Sulfur/paraffin; ground magnesium lime//polyol	Addition of hydrophobic sealant slows down release but still faster than commercial CRF.	NA	[33,34]
Phospho- gypsum	Paraffin wax/Span− 80	Addition of emulsifier significantly reduces the release rate due to enhanced paraffin adhesion.	10 days	[35]
**Mineral-based**				
Hydroxyapatite (HA)	Lignocellulosic biomass	Urea adsorption due to chemical bond with HA results in slow release. It can be further enhanced with the addition of hydrophobic filler.	5 min–3 days	[37,38]
Zeolite	Corn and potato starch, bentonite, white cement, acrylic polymer	Suitable binder type can slow down the release rate.	8 h (>8 h for acrylic polymer) ^b^	[39]
Bentonite	Starch, hydroxypropyl methylcellulose (HPMC); hydrophilic polymer (polyacrylamide); hydrophobic polymer (polycaprolactone)	Nanocomposite provides a superior controlled release. The urea release rate is affected by binder type and slowed down due to adsorption by bentonite.	2 days	[3,21,40]
Attapulgite (APT)	Ethyl cellulose (EC) and sodium carboxymethyl cellulose/hydroxyethyl cellulose hydrogel	Urea release slowed down due to adsorption by APT. Optimum. Carboxymethyl cellulose and hydroxymethyl cellulose (CMC/HEC) and crosslinker content are also important factors.	5 days	[41]

^a^ Time required to reach 75% release; ^b^ Release experiment only conducted until 40% release.

**Table 2:** Synthetic polymer-based CRFs.

Synthetic Polymer-Based				
Material	Modifier	Research Findings	Release Duration ^a^	References
Poly- styrene	Wax; Polyurethane	Wax is brittle and cannot prevent water penetration into the coating. Increasing size slows down release and reduces coating material required.	70 days	[43]
Poly- urethane (PU)	Mesoporous silica; Hydroxypropyl-terminated Polydimethylsiloxane (HP-PDMS)	Filler morphology affects the release rate. Implementation of hydrophobic gradient layer increases urea diffusion resistance.	55–70 days	[44,45]
Polyether sulfone	Fe_2_O_3_ nanoparticles (NPs)	A new class of CRF. Fe_2_O_3_ NP increases coating thickness and reduces release rate. It also allows the carrier to be recovered and recycled.	NA	[46]
**Biodegradable Synthetic Polymer-based**				
Aliphatic Polyester	-	The increasing size of CRF but using smaller urea crystals slows down degradability and release rate.	1 day	[47,48]
Bio-based Epoxy	Different ratio of liquified bagasse (LB) to bisphenol-A diglycidyl ether (BDE)	Optimum BDE amount increases compactness and hydrophobicity and retards release rate.	10–30 days	[49]
Polyvinyl Alcohol	PEG and Na_2_SO_4_; biochar	High water swelling rate and only 15–20% release on the first day. Improves water retention in soil and can adsorb Fe(III) ions which reduces toxicity to plants. Biochar improves mechanical strength, degradability and slows down release rate.	>30 days ^b^	[50,51]

^a^ Time required to reach 75% release; ^b^ Release experiment only conducted until 40% release.

**Table 3:** Natural polymer based CRFs.

Natural Polymer-Based				
Material	Modifier	Research Findings	Release Duration ^a^	References
Biobased Polyurethane (PU)	Isocyanate, acrylonitrile modification, superabsorbent from chicken feather meal; nano fumed silica	Double layer polymer coating significantly retards the release rate. Castor oil-based PU has better adherence as the coating material. Nano fumed silica reduces porosity and pore size. Isocyanate affects the structure of PU which affects the release rate.	14–77 days	[53,54,56,57,58,59]
Bio-based modified alkyd resin	Cassava Starch	Using castor oil reduces coating requirement compared to rubber oil.	NA	[55]
Polysulfone (SO_2_ and eugenol based)	-	Increasing M_w_ of polymer reduces the rate of degradation, slowing down the release.	3–30 days	[60]
Latex	-	Urea content affects swelling degree which greatly affects the release rate.	30 days	[61]
Natural rubber	Cassava starch; attapulgite/NR and NR-g-Polyacrylic acid	Hydrophobic NR can retard release rate with enhanced hydrophilicity through grafting. Multicoated CRF with NR and hydrogel shows great controlled release.	>24 h ^b^	[62,63]
Starch	bentonite; cellulose nanofibril from bagasse; natural char NP; bagasse, melamine, polyvinylacetate; EC	Urea can act as a plasticizer. Modification of starch to increase hydrophobicity and the use of reinforcing agent can improve controlled release. Starch-based hydrogel shows excellent water holding capacity and retention in soil. Using an appropriate filler creates interactions which slow down the release.	6–30 days	[4,64,65,66,67,68,69,70,71,72]
Cellulose	Silica NP, bentonite, montmorillonite (MMT)	Incorporation of filler into cellulose-based coating material promotes tortuous path and compactness which slows down diffusion.	6 days–30 days; >30 days (w/MMT)^c^	[73,74,75,76,77]
Lignin	Alkenyl succinic anhydride	Water-repelling properties shows great potential to retard nutrient release	10–30 min	[75]
Alginate	Κ-Carrageenan/celite superabsorbent; MMT; biogenic silica	Incorporation of filler increases porosity which improves water absorption and slows down the release.	18–50 days; >60 days (w/MMT)	[15,80,81]
Chitosan	Humic substances; starch+allicin; salicylaldehyde; magnesium+natural rubber	Smaller urea crystals can be better encapsulated in the matrix for slow release. Chitosan does not provide strong effects but incorporation with other materials may promote interactions that retard release.	7–13 days	[20,82,83,84]

^a^ Time required to reach 75% release; ^b^ Release experiment only conducted until 30% release; ^c^ Release experiment only conducted until 60.8% release.

**Table 4:** CRFs coating from other organic materials.

Other Organic Materials				
Material	Modifier	Research Findings	Release Duration ^a^	References
Biochar	Bentonite, sepiolite	Good urea sorption capability by biochar and mineral binder to slow down the release.	30 days	[31,86]
Rosin Adduct	Maleic anhydride	The effective barrier for urea release due to the covalent bond between maleic anhydride and urea. Works effectively under different soil texture.	4 days ^b^	[29]

^a^ Time required to reach 75% release; ^b^ Time required to release 45% and reached plateau.

**Table 5:** Summary of coating techniques.

Coating Techniques	Advantages	Disadvantages
*Physical Method*		
Rotary Drum	• Can be a continuous process, low operating cost, easily scaled	• Requires a large number of materials to achieve a uniform coating
Pan Coating	• Can be a continuous process, low operating cost, easily scaled	• High air temperature for drying • Poor maintenance of humidity level results in a defective structure
Fluidized Bed	• Can be a continuous process, low operating cost, easily scaled • Can achieve a more uniform coating • A more extensive selection of materials	• Expensive equipment • Long residence time • Prone to filter blocking • Higher chance of solvent explosion • Lower performance with larger granule size
Melting and Extrusion	• Solvent-free • Simple and cheap	• Hot melts are involved • Expensive equipment
*Chemical Method*		
Solution Polymerization/crosslinking	• Solvent reduces viscosity which makes it easier to process. • Crosslinking density can be controlled by varying monomer, initiator and cross-linking agent content	• Lower rate of reaction results in possible loss of compound • Difficult to recover solvent from its final form.
Inverse Suspension Polymerization	• Crosslinking density can be controlled by varying monomer, initiator and cross-linking agent content • Higher efficiency due to high reaction rate • The solvent can be recovered which reduces the cost	• Prone to contamination by the suspension • Must perform separation to purify polymer
Microwave Irradiation	• Simple and low energy consumption	• Not widely implemented in CRF preparation

## Data Availability

Not applicable.

## References

[1] HLEF Global Agriculture Towards 2050. http://www.fao.org/fileadmin/templates/wsfs/docs/Issues_papers/HLEF2050_Global_Agriculture.pdf.

[2] Ain N.U., Naveed M., Hussain A., Mumtaz M.Z., Rafique M., Bashir M.A., Alamri S., Siddiqui M.H. (2020). Impact of Coating of Urea with Bacillus-Augmented Zinc Oxide on Wheat Grown under Salinity Stress. Plants.

[3] Xiaoyu N., Yuejin W., Zhengyan W., Lin W., Guannan Q., Lixiang Y. (2013). A novel slow-release urea fertiliser: Physical and chemical analysis of its structure and study of its release mechanism. Biosyst. Eng..

[4] Versino F., Urriza M., García M.A. (2019). Eco-compatible cassava starch films for fertilizer controlled-release. Int. J. Biol. Macromol..

[5] Gil-Ortiz R., Naranjo M.Á., Ruiz-Navarro A., Caballero-Molada M., Atares S., García C., Vicente O. (2020). New Eco-Friendly Polymeric-Coated Urea Fertilizers Enhanced Crop Yield in Wheat. Agronomy.

[6] Savci S. (2012). Investigation of effect of chemical fertilizers on environment. APCBEE Procedia.

[7] Gil-Ortiz R., Naranjo M.Á., Ruiz-Navarro A., Atares S., García C., Zotarelli L., San Bautista A., Vicente O. (2020). Enhanced Agronomic Efficiency Using a New Controlled-Released, Polymeric-Coated Nitrogen Fertilizer in Rice. Plants.

[8] Messiga A.J., Dyck K., Ronda K., Van Baar K., Haak D., Yu S., Dorais M. (2020). Nutrients Leaching in Response to Long-Term Fertigation and Broadcast Nitrogen in Blueberry Production. Plants.

[9] Fan L.-T., Singh S.K., Fan L.-T., Singh S.K. (2012). Introduction. Controlled Release: A Quantitative Treatment.

[10] Law J.W.-F., Ser H.-L., Khan T.M., Chuah L.-H., Pusparajah P., Chan K.-G., Goh B.-H., Lee L.-H. (2017). The potential of Streptomyces as biocontrol agents against the rice blast fungus, Magnaporthe oryzae (Pyricularia oryzae). Front. Microbiol..

[11] Sun S., Tan L.T.-H., Fang Y.-L., Jin Z.-J., Zhou L., Goh B.-H., Lee L.-H., Zhou J., He Y.-W. (2020). Overexpression of oxyR Increases Phenazine-1-Carboxylic Acid Biosynthesis via Small RNA phrS in the Rhizobacterium Strain Pseudomonas PA1201. Mol. Plant Microbe Interact..

[12] Law J.W.-F., Chan K.-G., He Y.-W., Khan T.M., Ab Mutalib N.-S., Goh B.-H., Lee L.-H. (2019). Diversity of Streptomyces spp. from mangrove forest of Sarawak (Malaysia) and screening of their antioxidant and cytotoxic activities. Sci. Rep..

[13] Ser H.-L., Law J.W.-F., Tan W.-S., Yin W.-F., Chan K.-G., Lee L.-H. (2019). Genome sequence of bioactive streptomycete isolated from mangrove forest in East Malaysia, Streptomyces monashensis MUSC 1JT. Prog. Drug Discov. Biomed. Sci..

[14] Mangzira Kemung H., Tan L.T.-H., Chan K.-G., Ser H.-L., Law J.W.-F., Lee L.-H., Goh B.-H. (2020). Streptomyces sp. Strain musc 125 from mangrove soil in malaysia with anti-mrsa, anti-biofilm and antioxidant activities. Molecules.

[15] Wang Y., Liu M., Ni B., Xie L. (2012). κ-Carrageenan–sodium alginate beads and superabsorbent coated nitrogen fertilizer with slow-release, water-retention, and anticompaction properties. Ind. Chem. Eng. Res..

[16] Cole J.C., Smith M.W., Penn C.J., Cheary B.S., Conaghan K.J. (2016). Nitrogen, phosphorus, calcium, and magnesium applied individually or as a slow release or controlled release fertilizer increase growth and yield and affect macronutrient and micronutrient concentration and content of field-grown tomato plants. Sci. Hortic..

[17] Cong Z., Yazhen S., Changwen D., Jianmin Z., Huoyan W., Xiaoqin C. (2010). Evaluation of waterborne coating for controlled-release fertilizer using Wurster fluidized bed. Ind. Eng. Chem. Res..

[18] IFA Executive summary fertilizer outlook 2019–2023. Proceedings of the IFA Annual Conference.

[19] Wu Y. (2011). Chemical fertilizer use efficiency and its determinants in China’s farming sector. China Agric. Econ. Rev..

[20] Iftime M.M., Ailiesei G.L., Ungureanu E., Marin L. (2019). Designing chitosan based eco-friendly multifunctional soil conditioner systems with urea controlled release and water retention. Carbohydr. Polym..

[21] Hermida L., Agustian J. (2019). Slow release urea fertilizer synthesized through recrystallization of urea incorporating natural bentonite using various binders. Environ. Technol. Inno..

[22] Ravishankara A., Daniel J.S., Portmann R.W. (2009). Nitrous oxide (N2O): The dominant ozone-depleting substance emitted in the 21st century. Science.

[23] Trenkel M.E. (2010). Slow-and Controlled-Release and Stabilized Fertilizers: An Option for Enhancing Nutrient Use Efficiency in Agriculture.

[24] Shaviv A. Controlled release fertilizers. Proceedings of the IFA International Workshop on Enhanced-Efficiency Fertilizers.

[25] AAPFCO (1995). Official Publication No. 48.

[26] Shaviv A. (2001). Advances in controlled-release fertilizers. Adv. Agron..

[27] Sempeho S.I., Kim H.T., Mubofu E., Hilonga A. (2014). Meticulous overview on the controlled release fertilizers. Adv. Chem..

[28] Lammel J. Cost of the different options available to the farmers: Current situation and prospects. Proceedings of the IFA International Workshop on Enhanced-Efficiency Fertilizers.

[29] Mumtaz I., Majeed Z., Ajab Z., Ahmad B., Khurshid K., Mubashir M. (2019). Optimized tuning of rosin adduct with maleic anhydride for smart applications in controlled and targeted delivery of urea for higher plant’s uptake and growth efficiency. Ind. Crops. Prod..

[30] Wang Y., Guo H., Wang X., Ma Z., Li X., Li R., Li Q., Wang R., Jia X. (2020). Spout Fluidized Bed Assisted Preparation of Poly (tannic acid)-Coated Urea Fertilizer. ACS Omega.

[31] Shi W., Ju Y., Bian R., Li L., Joseph S., Mitchell D.R., Munroe P., Taherymoosavi S., Pan G. (2020). Biochar bound urea boosts plant growth and reduces nitrogen leaching. Sci. Total Environ..

[32] Vashishtha M., Dongara P., Singh D. (2010). Improvement in properties of urea by phosphogypsum coating. Int. J. Chemtech. Res..

[33] Ibrahim K.R.M., Babadi F.E., Yunus R. (2014). Comparative performance of different urea coating materials for slow release. Particuology.

[34] Babadi F.E., Yunus R., Rashid S.A., Salleh M.A.M., Ali S. (2015). New coating formulation for the slow release of urea using a mixture of gypsum and dolomitic limestone. Particuology.

[35] Yu X., Li B. (2019). Release mechanism of a novel slow-release nitrogen fertilizer. Particuology.

[36] Dixon J. (1991). Roles of clays in soils. Appl. Clay. Sci..

[37] Kottegoda N., Sandaruwan C., Priyadarshana G., Siriwardhana A., Rathnayake U.A., Berugoda Arachchige D.M., Kumarasinghe A.r., Dahanayake D., Karunaratne V., Amaratunga G.A. (2017). Urea-hydroxyapatite nanohybrids for slow release of nitrogen. ACS Nano.

[38] Elhassani C.E., Essamlali Y., Aqlil M., Nzenguet A.M., Ganetri I., Zahouily M. (2019). Urea-impregnated HAP encapsulated by lignocellulosic biomass-extruded composites: A novel slow-release fertilizer. Environ. Technol. Inno..

[39] Dubey A., Mailapalli D.R. (2019). Zeolite coated urea fertilizer using different binders: Fabrication, material properties and nitrogen release studies. Environ. Technol. Inno..

[40] Pereira E.I., Da Cruz C.C., Solomon A., Le A., Cavigelli M.A., Ribeiro C. (2015). Novel slow-release nanocomposite nitrogen fertilizers: The impact of polymers on nanocomposite properties and function. Ind. Chem. Eng. Res..

[41] Ni B., Liu M., Lu S., Xie L., Wang Y. (2011). Environmentally friendly slow-release nitrogen fertilizer. J. Agric. Food. Chem..

[42] Azeem B., Kushaari K., Man Z.B., Basit A., Thanh T.H. (2014). Review on materials & methods to produce controlled release coated urea fertilizer. J. Control. Release.

[43] Yang Y.-C., Zhang M., Li Y., Fan X.-H., Geng Y.-Q. (2012). Improving the quality of polymer-coated urea with recycled plastic, proper additives, and large tablets. J. Agric. Food. Chem..

[44] Li L., Sun Y., Cao B., Song H., Xiao Q., Yi W. (2016). Preparation and performance of polyurethane/mesoporous silica composites for coated urea. Mater. Des..

[45] Dai C., Yang L., Xie J., Wang T.-J. (2020). Nutrient diffusion control of fertilizer granules coated with a gradient hydrophobic film. Colloids Surf. A.

[46] Emami N., Razmjou A., Noorisafa F., Korayem A.H., Zarrabi A., Ji C. (2017). Fabrication of smart magnetic nanocomposite asymmetric membrane capsules for the controlled release of nitrate. Environ. Nanotechnol. Monit. Manag..

[47] Ye H.-M., Li H.-F., Wang C.-S., Yang J., Huang G., Meng X., Zhou Q. (2020). Degradable polyester/urea inclusion complex applied as a facile and environment-friendly strategy for slow-release fertilizer: Performance and mechanism. Chem. Eng. J..

[48] Bi S., Barinelli V., Sobkowicz M.J. (2020). Degradable Controlled Release Fertilizer Composite Prepared via Extrusion: Fabrication, Characterization, and Release Mechanisms. Polymers.

[49] Li Y., Jia C., Zhang X., Jiang Y., Zhang M., Lu P., Chen H. (2018). Synthesis and performance of bio-based epoxy coated urea as controlled release fertilizer. Prog. Org. Coat.

[50] Sarkar K., Sen K. (2018). Polyvinyl alcohol based hydrogels for urea release and Fe (III) uptake from soil medium. J. Environ. Chem. Eng..

[51] Chen S., Yang M., Ba C., Yu S., Jiang Y., Zou H., Zhang Y. (2018). Preparation and characterization of slow-release fertilizer encapsulated by biochar-based waterborne copolymers. Sci. Total. Environ..

[52] Lubkowski K., Smorowska A., Grzmil B., Kozłowska A. (2015). Controlled-release fertilizer prepared using a biodegradable aliphatic copolyester of poly (butylene succinate) and dimerized fatty acid. J. Agric. Food. Chem..

[53] Yang Y., Tong Z., Geng Y., Li Y., Zhang M. (2013). Biobased polymer composites derived from corn stover and feather meals as double-coating materials for controlled-release and water-retention urea fertilizers. J. Agric. Food. Chem..

[54] Bortoletto-Santos R., Ribeiro C., Polito W.L. (2016). Controlled release of nitrogen-source fertilizers by natural-oil-based poly (urethane) coatings: The kinetic aspects of urea release. J. Appl. Polym. Sci..

[55] Uzoh C.F., Onukwuli O.D., Ozofor I.H., Odera R.S. (2019). Encapsulation of urea with alkyd resin-starch membranes for controlled N2 release: Synthesis, characterization, morphology and optimum N2 release. Process. Saf. Environ..

[56] Bortoletto-Santos R., Cavigelli M.A., Montes S.E., Schomberg H.H., Le A., Thompson A.I., Kramer M., Polito W.l., Ribeiro C. (2020). Oil-based polyurethane-coated urea reduces nitrous oxide emissions in a corn field in a Maryland loamy sand soil. J. Clean. Prod..

[57] Liu L., Shen T., Yang Y., Gao B., Li Y.C., Xie J., Tang Y., Zhang S., Wang Z., Chen J. (2018). Bio-based Large Tablet Controlled-Release Urea: Synthesis, Characterization, and Controlled-Released Mechanisms. J. Agric. Food Chem..

[58] Dong Feng G., Ma Y., Zhang M., You Jia P., Hong Hu L., Guo Liu C., Hong Zhou Y. (2019). Polyurethane-coated urea using fully vegetable oil-based polyols: Design, nutrient release and degradation. Prog. Org. Coat.

[59] Liu J., Yang Y., Gao B., Li Y.C., Xie J. (2019). Bio-based elastic polyurethane for controlled-release urea fertilizer: Fabrication, properties, swelling and nitrogen release characteristics. J. Clean. Prod..

[60] Liu L., Ni Y., Zhi Y., Zhao W., Pudukudy M., Jia Q., Shan S., Zhang K., Li X. (2020). Sustainable and Biodegradable Copolymers from SO_2_ and Renewable Eugenol: A Novel Urea Fertilizer Coating Material with Superio Slow Release Performance. Macromolecules.

[61] Yang L., An D., Wang T.-J., Kan C., Jin Y. (2017). Swelling and diffusion model of a hydrophilic film coating on controlled-release urea particles. Particuology.

[62] Riyajan S.-A., Sasithornsonti Y., Phinyocheep P. (2012). Green natural rubber-g-modified starch for controlling urea release. Carbohydr. Polym..

[63] Cui Y., Xiang Y., Xu Y., Wei J., Zhang Z., Li L., Li J. (2020). Poly-acrylic acid grafted natural rubber for multi-coated slow release compound fertilizer: Preparation, properties and slow-release characteristics. Int. J. Biol. Macromol..

[64] Rychter P., Kot M., Bajer K., Rogacz D., Sišková A., Kapuśniak J. (2016). Utilization of starch films plasticized with urea as fertilizer for improvement of plant growth. Carbohydr. Polym..

[65] Niu Y., Li H. (2012). Controlled release of urea encapsulated by starch-g-poly (vinyl acetate). Ind. Eng. Chem. Res..

[66] Giroto A.S., Guimarães G.G., Colnago L.A., Klamczynski A., Glenn G., Ribeiro C. (2019). Controlled release of nitrogen using urea-melamine-starch composites. J. Clean. Prod..

[67] Jin S., Wang Y., He J., Yang Y., Yu X., Yue G. (2013). Preparation and properties of a degradable interpenetrating polymer networks based on starch with water retention, amelioration of soil, and slow release of nitrogen and phosphorus fertilizer. J. Appl. Polym. Sci..

[68] Xiao X., Yu L., Xie F., Bao X., Liu H., Ji Z., Chen L. (2017). One-step method to prepare starch-based superabsorbent polymer for slow release of fertilizer. Chem. Eng. J..

[69] Wen P., Han Y., Wu Z., He Y., Ye B.-C., Wang J. (2017). Rapid synthesis of a corncob-based semi-interpenetrating polymer network slow-release nitrogen fertilizer by microwave irradiation to control water and nutrient losses. Arab. J. Chem..

[70] Salimi M., Motamedi E., Motesharezedeh B., Hosseini H.M., Alikhani H.A. (2020). Starch-g-poly (acrylic acid-co-acrylamide) composites reinforced with natural char nanoparticles toward environmentally benign slow-release urea fertilizers. J. Environ. Chem. Eng..

[71] Qiao D., Liu H., Yu L., Bao X., Simon G.P., Petinakis E., Chen L. (2016). Preparation and characterization of slow-release fertilizer encapsulated by starch-based superabsorbent polymer. Carbohydr. Polym..

[72] Patil M.D., Patil V.D., Sapre A.A., Ambone T.A., Torris At A., Shukla P.G., Shanmuganathan K. (2018). Tuning controlled release behavior of starch granules using nanofibrillated cellulose derived from waste sugarcane bagasse. ACS Sustain. Chem. Eng..

[73] Xie L., Liu M., Ni B., Wang Y. (2012). Utilization of wheat straw for the preparation of coated controlled-release fertilizer with the function of water retention. J. Agric. Food. Chem..

[74] Li X., Li Q., Su Y., Yue Q., Gao B., Su Y. (2015). A novel wheat straw cellulose-based semi-IPNs superabsorbent with integration of water-retaining and controlled-release fertilizers. J. Taiwan Inst. Chem. Eng..

[75] Bortolin A., Aouada F.A., Mattoso L.H., Ribeiro C. (2013). Nanocomposite PAAm/methyl cellulose/montmorillonite hydrogel: Evidence of synergistic effects for the slow release of fertilizers. J. Agric. Food. Chem..

[76] Wen P., Wu Z., He Y., Ye B.-C., Han Y., Wang J., Guan X. (2016). Microwave-assisted synthesis of a semi-interpenetrating polymer network slow-release nitrogen fertilizer with water absorbency from cotton stalks. ACS Sustain. Chem. Eng..

[77] Olad A., Zebhi H., Salari D., Mirmohseni A., Tabar A.R. (2018). Slow-release NPK fertilizer encapsulated by carboxymethyl cellulose-based nanocomposite with the function of water retention in soil. Mater. Sci. Eng. C.

[78] Mulder W., Gosselink R., Vingerhoeds M., Harmsen P., Eastham D. (2011). Lignin based controlled release coatings. Ind. Crops. Prod..

[79] Tønnesen H.H., Karlsen J. (2002). Alginate in drug delivery systems. Drug Dev. Ind. Pharm..

[80] Rashidzadeh A., Olad A. (2014). Slow-released NPK fertilizer encapsulated by NaAlg-g-poly (AA-co-AAm)/MMT superabsorbent nanocomposite. Carbohydr. Polym..

[81] De Matos M., Mattos B.D., Tardy B.L., Rojas o.J., Magalhães W.L. (2018). Use of Biogenic Silica in Porous Alginate Matrices for Sustainable Fertilization with Tailored Nutrient Delivery. ACS Sustain. Chem. Eng..

[82] Araújo B.R., Romão L.P., Doumer M.E., Mangrich A.S. (2017). Evaluation of the interactions between chitosan and humics in media for the controlled release of nitrogen fertilizer. J. Environ. Manag..

[83] Huey C.E., Yahya W.Z.N., Mansor N. Allicin incorporation as urease inhibitor in a chitosan/starch based biopolymer for fertilizer application. Proceedings of the Conference on Biomedical and Advanced Materials, The Bayview Hotel Langkawi.

[84] Adlim M., Zarlaida F., Rahmayani R.F.I., Wardani R. (2019). Nutrient release properties of a urea–magnesium–natural rubber composite coated with chitosan. Environ. Technol. Innov..

[85] Yang D., Yunguo L., Shaobo L., Huang x., Zhongwu L., Xiaofei T., Guangming Z., Lu Z. (2017). Potential benefits of biochar in agricultural soils: A review. Pedosphere.

[86] Wen P., Wu Z., Han Y., Cravotto G., Wang J., Ye B.-C. (2017). Microwave-assisted synthesis of a novel biochar-based slow-release nitrogen fertilizer with enhanced water-retention capacity. ACS Sustain. Chem. Eng..

[87] Sun Y.-M., Huang W.-F., Chang C.-C. (1999). Spray-coated and solution-cast ethylcellulose pseudolatex membranes. J. Membr. Sci..

[88] Beig B., Niazi M.B.K., Jahan Z., Hussain A., Zia M.H., Mehran M.T. (2020). Coating materials for slow release of nitrogen from urea fertilizer: A review. J. Plant. Nutr..

[89] Hede P.D., Bach P., Jensen A.D. (2009). Fluidized-Bed Coating with Sodium Sulfate and PVA− TiO2, 1. Review and Agglomeration Regime Maps. Ind. Eng. Chem. Res..

[90] Nickerson M., Yan C., Cloutier S., Zhang W., Gaonkar A.G., Vasisht N., Khare A.R., Sobel R. (2014). Protection and masking of omega-3 and-6 oils via microencapsulation. Microencapsulation in the Food Industry.

[91] Teunou E., Poncelet D. (2002). Batch and continuous fluid bed coating–review and state of the art. J. Food Eng..

[92] Popov B.N., Popov B.N. (2015). Organic coatings. Corrosion Engineering: Principles and Solved Problems.

[93] Uzma N., Khaja Mohinuddin Salar B., Kumar B.S., Aziz N., David M.A., Reddy V.D. (2008). Impact of organic solvents and environmental pollutants on the physiological function in petrol filling workers. Int. J. Environ. Res..

[94] Lokensgard E. (2016). Industrial Plastics: Theory and Applications.

[95] Ahmed E.M. (2015). Hydrogel: Preparation, characterization, and applications: A review. J. Adv. Res..

[96] Ibrahim S., Nawwar G.A., Sultan M. (2016). Development of bio-based polymeric hydrogel: Green, sustainable and low cost plant fertilizer packaging material. J. Environ. Chem. Eng..

[97] Du C.-W., Zhou J.-M., Shaviv A. (2006). Release characteristics of nutrients from polymer-coated compound controlled release fertilizers. J. Polym. Environ..

[98] Basu S., Kumar N., Srivastava J. (2010). Modeling NPK release from spherically coated fertilizer granules. Simul. Model. Pract. Theory.

[99] Shaviv A., Raban S., Zaidel E. (2003). Modeling controlled nutrient release from polymer coated fertilizers: Diffusion release from single granules. Environ. Sci. Technol..

[100] Irfan S.A., Razali R., Kushaari K., Mansor N., Azeem B., Versypt A.N.F. (2018). A review of mathematical modeling and simulation of controlled-release fertilizers. J. Control. Release.

[101] Bruschi M.L., Bruschi M.L. (2015). Mathematical and physiochemical models of drug release. Strategies to Modify the Drug Release from Pharmaceutical Systems.

[102] Jarrell W., Boersma L. (1980). Release of urea by granules of sulfur-coated urea. Soil Sci. Soc. Am. J..

[103] Glasser V., Stajer P., Vidensky J., Svandova P., Knor V. (1987). Urea-formaldehyde resins as packaging materials for industrial fertilisers with protracted action. Part 4. Int. Polym. Sci. Technol..

[104] Lu S., Lee S. (1992). Slow release of urea through latex film. J. Control. Release.

[105] Lu S., Chang S.-L., Ku W.-Y., Chang H.-C., Wang J.-Y., Lee D.-J. (2007). Urea release rate from a scoop of coated pure urea beads: Unified extreme analysis. J. Chin. Inst. Chem. Eng..

[106] Trinh T.H., Kushaari K., Basit A., Azeem B., Shuib A. (2014). Use of multi-diffusion model to study the release of urea from urea fertilizer coated with polyurethane-like coating (PULC). APCBEE Procedia.

[107] Shen Y.Z., Du C.W., Zhou J.M., Ma F. (2015). Modeling Nutrient Release fromSwelling Polymer-Coated Urea. Appl. Eng. Agric..

[108] Shaviv A., Raban S., Zaidel E. (2003). Modeling controlled nutrient release from a population of polymer coated fertilizers: Statistically based model for diffusion release. Environ. Sci. Technol..

[109] Trinh T.H., Kushaari K., Shuib A.A., Ismail L., Azeem B. (2015). Modelling the release of nitrogen from controlled release fertiliser: Constant and decay release. Biosyst. Eng..

[110] Trinh T.H., Kushaari K., Basit A. (2015). Modeling the release of nitrogen from controlled-release fertilizer with imperfect coating in soils and water. Ind. Chem. Eng. Res..

[111] Du C., Tang D., Zhou J., Wang H., Shaviv A. (2008). Prediction of nitrate release from polymer-coated fertilizers using an artificial neural network model. Biosyst. Eng..

[112] Ambrose R.P.K., Wassgren C.R., Pai D., Chen Y. (2020). Layer-Wise Agglomerated Urea Granules. U.S. Patent.

[113] Adhikari R., Muster T.H., Freischmidt G. (2020). Controlled Release Granular Fertiliser. U.S. Patent.

[114] Li J., Jingling Z., Song X., Ong C.N., Loh C.S., Tan W.K. (2020). Production of Nutrigel Materials from Soya Waste. U.S. Patent.

[115] Kannan G., Posada C., Haigh J., Harper T., Kanagalingam S. (2019). Coated Granular Fertilizers, Methods of Manufacture Thereof, and Uses Thereof. U.S. Patent.

[116] Flores J., Bosley M.A., Rifai S., Mahoney R.P., Hajduk D., Casado Portilla R., Newton T., Soane D.S. (2020). Nontoxic Coating Concentrates for Agricultural Uses. U.S. Patent.

[117] Venkatramesh M., Kendirgi F., Sanders S.D., Sanders A.Z., Hasinoff M.P., Pursell J.T. (2020). Microbial Coating of Controlled-Release Fertilizers. U.S. Patent.

[118] Nave B., Pasda G., Wissemeier A., Staal M., Schneider K.-H., Schmid M., Zerulla W., Lohe D., Zhu S.S. (2020). Mixtures Comprising a Biopesticide and a Nitrification Inhibitor. U.S. Patent.

